# Supplementary material for: Chromothripsis during telomere crisis is independent of NHEJ, and consistent with a replicative origin
Source: Genome Res. 2019 May;29(5):737–49. doi: 10.1101/gr.240705.118 (PMC6499312; doi:10.1101/gr.240705.118)
Supplement: Supplemental Material [file supp_gr.240705.118_Supplemental_file_1.zip › contigs/annotated_contigs/DB80/contig.2.DB80_length_535_mean_cov_8.97196261682.docx]

**DB80_length_535_mean_cov_8.97196261682**

```
TGGGCAGAAAAATGAGGGGATAAGAATCCAACCTGAGGGAACAAGACAATTTCTGGGAATTGCCAACAGTTTTTAGAAGAGAGTCATGG
 >chr3:47339477-47339740 + E=1e-146 p=0e+00
TGGGGGATAAAACTGGATGGGGGCCAGGTGCAGCGGCTCACACCTATAATCCCAGCACTTTGGGAGGCTGATGCAGAAGGAAGCCTTAA

ATCCAGGAGTTCAAGACCAGCCTGGACAACAAAGTGAGACCCCATCTCTACCAAAAATAAAAAAATTGGCCAGGTACCTAT|AGTC|TA
                                                                                  >chr3
GATGTTGCTGTGAAGATATTTTTTAGATGTGATTAACATTTAAATTAGTAGACTTTAGTTGAGTACAGTGGTATGTGCCTGTAATCCCA
:47340704-47340980 + E=2e-154
GCTACCTGGGAAGCTGAGGCGGGAGGATTGCTTGAGCCCAGGAGTCTGAAACCAACTTGGGCAACATAGTGAGACCCCATCTCATTTTA

AAATAATAATAAGTTAGTAGACTTTCAGTAAAGTTGATTACCTTCCATAATGTGGGTGGGCCTCATCCAGTCAGTGTAAGGCCTTAAAG

AGC
```
